# Comprehensive Assessment of the Stability of Selected Coxibs in Variable Environmental Conditions along with the Assessment of Their Potential Hepatotoxicity

**DOI:** 10.3390/pharmaceutics15112609

**Published:** 2023-11-09

**Authors:** Paweł Gumułka, Łukasz Pecio, Paweł Żmudzki, Krzesimir Ciura, Krystyna Skalicka-Woźniak, Monika Dąbrowska, Małgorzata Starek

**Affiliations:** 1 Doctoral School of Medical and Health Sciences, Jagiellonian University Medical College, 16 Łazarza St., 31-530 Kraków, Poland; pawel.gumulka@student.uj.edu.pl; 2 Department of Inorganic and Analytical Chemistry, Faculty of Pharmacy, Jagiellonian University Medical College, 9 Medyczna St., 30-688 Kraków, Poland; monika.1.dabrowska@uj.edu.pl; 3 Department of Chemistry of Natural Products, Medical University of Lublin, 1 Chodźki St., 20-093 Lublin, Poland; lukaszpecio@umlub.pl (Ł.P.); kskalicka@pharmacognosy.org (K.S.-W.); 4 Department of Medicinal Chemistry, Faculty of Pharmacy, Jagiellonian University Medical College, 9 Medyczna St., 30-688 Kraków, Poland; pawel.zmudzki@uj.edu.pl; 5 Department of Physical Chemistry, Medical University of Gdańsk, Aleja Gen. Hallera 107, 80-416 Gdańsk, Poland; krzesimir.ciura@gumed.edu.pl

**Keywords:** coxibs, degradation study, TLC-densitometry, hepatotoxicyty, UPLC-MS/MS, chemometry, celecoxib, etoricoxib, firocoxib, robenacoxib, cimicoxib

## Abstract

Determining the influence of environmental factors on the stability of drugs is very helpful when choosing excipients, storage conditions or packaging materials. In addition, information about possible toxic degradation products enables detecting and avoiding the harmful side effects of the drug. We used the thin-layer chromatographic-densitometric procedure for the assay of five coxibs, conducted degradation studies in various environments and at different temperatures along with the determination of pharmacokinetic parameters. The results were subjected to chemometric analysis, to investigate and visualize the similarities and differences of the studied coxibs. Samples of the tested drug were also analyzed by UPLC-MS/MS in order to identify degradation products, and determine possible drug degradation pathways. Using the human liver cancer HepG2 cell line, the hepatotoxic effect of the degradation products was also determined. It was observed that all substances were relatively stable under the analyzed conditions and degraded more in acidic than alkaline environments. Robenacoxib is the drug that decomposes the fastest, and cimicoxib turned out to be the most stable. Robenacoxib also showed significant hepatotoxicity at the highest tested concentration, which correlates with the high degree of its degradation, and the probable formation of a more hepatoxic product. The obtained mass spectra of compounds formed as a result of hydrolysis of the protonated drug leading to the formation of several product ions, which enabled us to propose probable degradation pathways.

## 1. Introduction

Non-steroidal anti-inflammatory drugs (NSAIDs) are one of the most frequently used groups of drugs in the world. The mechanism of their action is based on the inhibition of the enzyme cyclooxygenase (COX), which is responsible for the conversion of arachidonic acid into signaling molecules, prostaglandins [1,2]. Over the years, at least three isoforms of this enzyme have been proven to exist. COX-1 is present in most tissues, and is responsible for maintaining homeostasis, monitoring and regulating the normal physiological conditions of the body. It is involved in general body processes such as cytoprotection of the gastric mucosa, platelet aggregation, vascular smooth muscle function, regulation of glomerular filtration and renal blood flow. COX-2 in the adult is found exclusively in the central nervous system (CNS), kidneys, alveoli, and placenta, while it is found in the heart, kidneys, lungs, and skin in the fetus. This form is highly induced by various factors, such as growth factor or cytokines, and is responsible for mediating pain and fever, and changes in various pathological and physiological states [3,4,5]. The beneficial analgesic, anti-inflammatory and antipyretic effects were thought to be primarily due to inhibition of the inducible form of the enzyme known as COX-2. Disrupting of natural processes by inhibiting COX-1 can cause many adverse effects, most commonly gastrotoxicity. Currently, works are progressing on the synthesis of more selective COX-2 inhibitors. ‘Coxib’ drugs are considered to be the most selective for the COX-2 enzyme isoform, which reduces gastrointestinal toxicity [6,7,8,9,10,11]. In 2002, the COX-3, a variant of COX-1, was discovered by post-transcriptional modification of COX-1 mRNA. COX-3 occurs, among others, in the structures of the CNS, and its activity is inhibited, e.g., by paracetamol, metamizole or diclofenac [12].

There are currently two selective COX-2 inhibitors in clinical use in humans—celecoxib and etoricoxib—in various indications as analgesic and anti-inflammatory drugs. Moreover, several drugs from this group were developed, but they were quickly withdrawn from the market (rofecoxib, valdecoxib, and lumiracoxib). The reason for this way was, e.g., increased cardiotoxicity or severe hepatotoxicity, which was found in clinical trials [13,14,15,16]. Many more preparations with a selective effect on the COX-2 enzyme, e.g., cimicoxib, firocoxib, and robenacoxib, are currently used in veterinary medicine. According to the latest research, these drugs seem to be a good solution in the treatment of ailments related to joint degeneration or post-operative pain. The cardiotoxic effect of these drugs has not been unequivocally proven. Many classic NSAIDs are not effective or cannot be used in certain animal species. Attention should also be paid to the possibility that animals are more sensitive to coxibs than humans due to differences in metabolism, absorption and elimination of the drug [17,18,19,20,21,22,23,24,25,26,27,28].

Changes occurring during exposure of the active medicinal substance (API) to environmental factors are very important both for determining the form of the drug, and its durability and stability. Moreover, information about possible degradation products enables the detection of harmful side effects already at an early stage of the research. Identifying structures, pathways, and degradation mechanisms under different environmental conditions is crucial to understanding the API profile. The obtained information can be used to improve the API production process, and select appropriate excipients, packaging materials and storage conditions [29]. There are several studies available, in which the degradation for a representative of the coxib group, etoricoxib, was carried out in both an acidic and alkaline environment [30,31,32,33]. Other studies present the analysis of celecoxib degradation in various environments [34,35,36]. A forced degradation trial of firocoxib under different stress conditions (acidic, alkaline, oxidative, thermal and photoinduced) was conducted, as recommended in the ICH guidelines [37]. Most of the available papers are aimed at showing a given method as capable of determining the active substance along with its decomposition products. Only some studies focused on quantitative changes and stability of celecoxib and etoricoxib. A review of the available literature shows that for the other two coxibs, cimicoxib and robenacoxib, degradation studies in different environments have not been performed.

**Figure 1:**
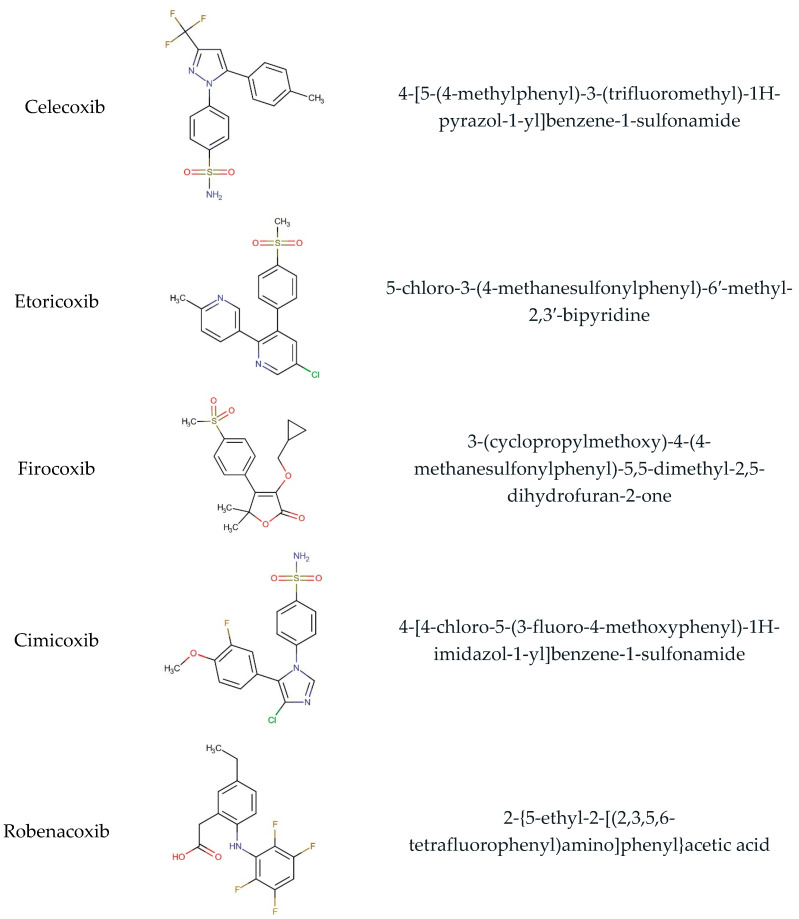
Chemical structure and IUPAC names of analyzed compounds (DrugBank Online; drugbank.com (accessed on 4 September 2023)).

Our team developed and validated the analytical procedure for the simultaneous determination of five coxibs, celecoxib (CEL), etoricoxib (ETO), firocoxib (FIR), robenacoxib (ROF) and cimicoxib (CIM) (Figure 1) [38]. In the presented study, the elaborated method was used to investigate the degradation process and kinetics of the decomposition of the tested five coxib in various environments (acidic, neutral and alkaline conditions) and at different temperatures. We analyzed the obtained results to determine existing relationships using statistical and chemometric methods. In addition, an attempt was made to define the structure of degradation products formed in the case of robenacoxib, which showed the lowest stability under the analyzed conditions. We also conducted a cell metabolic activity test (MTT) to check the potential hepatotoxicity of the tested substances, and their mixtures with decomposition products.

## 2. Materials and Methods

### 2.1. Materials

Methanol, chloroform, acetone, toluene and other organic solvents were purchased from Merck (Darmstadt, Germany). All chemicals were an analytical grade. Hydrochloric acid and sodium hydroxide solutions were purchased from POCh (Gliwice, Poland). Buffer solutions (pH 2.0—citrate buffer, pH 7.0—phosphate buffer, pH 9.2—borate buffer) were purchased from Witko (Łódź, Poland). Densitometer TLC Scanner 3 with winCat4 software ver.1.4.1 (CAMAG, Muttenz, Switzerland), Linomat V (CAMAG, Muttenz, Switzerland) and analytical balance XA 52/Y (Radwag, Poland) were used. Chromatographic plates HPTLC Silica gel 60F_254_ (no. 1.05548) were purchased from Merck (Darmstadt, Germany).

### 2.2. Standard Substances

Celecoxib (no. PHR1683), etoricoxib (no. 32097) and firocoxib (no. 32236) were purchased from Sigma-Aldrich (Burlington, MA, USA). Cimicoxib (no. C441690) and robenacoxib (no. R638000) were purchased from TRC Canada (Toronto Research Chemicals Inc., Toronto, ON, Canada). Pharmaceutical preparations: Celebrex (Pfizer Europe, Sandwich, UK) capsules containing 200 mg of CEL, Arcoxia (MSD, Warsaw, Poland) tablets containing 120 mg of ETO, Previcox (Merial, Lyon, France) chewable tablets containing 227 mg of FIR, Cimalgex (Vétoquinol SA, Lure, France) chewable tablets containing 80 mg of CIM, and Onsior (Elanco GmbH, Coxhaven, Germany) tablets containing 40 mg of ROB, were used. All preparations were purchased from the local pharmacy or veterinary office. Preparations had an expiry of not less than one year at the time of study.

### 2.3. Sample Solutions

Samples solutions were prepared by weighed, to an accuracy of 0.1 mg, a quantity of drug corresponding to 10.0 mg of each coxib. Drugs were transferred separately into a 10.0 mL volumetric flasks containing approximately 5.0 mL of methanol. Each sample was shaken on a laboratory shaker (Lab Dancer V Vario, Imlab, Boutersem, Belgum) for 5 min, and then the volume was topped up to 10.0 mL with methanol. The prepared solutions were used for the further analysis.

### 2.4. Chromatographic Conditions

Chromatography was performed on 10 × 10 cm aluminum sheets precoated with silica gel 60 F_254_. Samples were applied to the plates as bands 5 mm wide and 10 mm apart, using a Linomat V (sample applicator) equipped with a 100 μL syringe (Hamilton, Sigma-Aldrich, Poznań, Poland). The first application was 10 mm from the bottom and 10 mm from the left edge of the plate. The application rate was 600 nL/s. Plates were taken into a chromatographic chamber (18 × 16 × 8 cm; Sigma-Aldrich) previously saturated with mobile phase vapor for 20 min at room temperature. Good separation and well-developed peaks were obtained with mobile phase containing chloroform: acetone: toluene (12:5:2, *v*/*v*/*v*). The development distance was 10 cm, during 20 min. After development, plates were dried at room temperature for approximately 20 min. Densitometric scanning was performed using a TLC Scanner 3 with winCats 4 software ver.1.4.1. The source of radiation was the deuterium lamp emitting a continuous UV spectrum between 200 to 400 nm. Scanning speed was 20 mm/s, and slit dimensions were 4.00 × 0.45 mm. Based on the obtained spectra, analytical wavelengths were chosen for the coxibs detection: 254 nm for CEL and CIM, and 290 nm for ETO, FIR and ROB.

### 2.5. Degradation Study

The effect of pH, temperature and incubation time on the stability of five analyzed coxibs in solutions was investigated. For this purpose, amounts of approximately 20 mg of each standard substance were weighed, and each of the reagents (1 M HCl, 0.5 M HCl, distilled water, 1 M NaOH, 0.5 M NaOH, buffer pH 2.0, buffer pH 7.0, buffer pH 9.2) was added successively into a volume of 10 mL. The solutions were incubated (POL-EKO ILP53 Smart incubator; WITKO, Łódź, Poland) at room temperature (23 °C for 90 days), 70 °C (for 16 days) and 120 °C (for 130 h), and then collected at the time intervals specified in the study design. Next, samples were diluted with methanol (1:1, *v*/*v*) to give a final concentration of 10 mg/mL, and subjected to further analysis. The obtained solutions were applied to TLC plates in triplicate, and separation was conducted according to the method described above.

The degradation process of tested coxibs was evaluated by basic kinetic and thermodynamic parameters. The reaction rate k, the half-life time t_0.5_ (the time after which 50% of the substance was degraded), t_0.1_ (the time taken for 10% of the substance to decompose), activation energy E_a_, enthalpy ∆H* and entropy of activation ∆S* were calculated [39]. The obtained data enabled determining the rate of reactions, as proceeding according to first-order kinetics. The indicated parameters were calculated from the formulas:

k = 2.303 × (log(C_0_/C_t_)/t)

t_0.5_ = 0.693 × k

t_0.1_ = 0.1053 × k

E_a_ = [−2.303 × R × (logk_1_ − logk_2_)]/(1/T_1_ − 1/T_2_)

∆H* = E_a_ − R × T

∆S* = −∆H*/T

∆G = ∆H − T∆S

where t—time, C_0_—initial concentration, C_t_—concentration remaining after time interval t, k_2_ > k_1_, T_2_ > T_1_, R—gas constant (8.315 J/mol∙K), T—temperature [K], and G—Gibbs free energy.

### 2.6. Chemometric Analysis

Principal component analysis (PCA), heatmap, and hierarchical analysis (HCA) were performed using RStudio software (v. 2023.03.0), based on the data matrix of the remaining percentage of coxibs after forces degradation. Euclidian distance measure and a complete clustering method were chosen for HCA analysis. All the above-mentioned methods were applied to study similarities and dissimilarities of target coxibs in the designed space of degradation experiments.

### 2.7. Hepatotoxicity Tests

Human liver cancer HepG2 cell line (HB-8065™, ATCC, Manassas, VA, USA) cells were grown in culture flasks under standard conditions of temperature (37 °C) and CO_2_ concentration (5%). According to manufacturer procedures, cells were cultured in Eagle’s Minimum Essential Medium (EMEM, ATCC, Manassas, VA, USA) supplemented with 10% fetal bovine serum (FBS; Gibco, Waltham, MA, USA) and antibiotics (Gibco, Waltham, MA, USA). The experiment cells were seeded on 96-well plates at a density of 10,000/well. After 24 h, cells were incubated with a solution after coxibs water degradation (ROB at 120 °C for 8 h, and ROB, CEL, ETO, FIR, CIM at 70 °C for 7 days), at a final concentration of 3.125 to 50 μg/mL. After 48 h of incubation, the medium containing tested compounds was removed, and replaced by the fresh medium containing the MTT (3-(4,5-dimethylthiazol-2-yl)-2,5-diphenyltetrazolium bromide; Sigma-Aldrich, Darmstadt, Germany) (5 mg/mL). After 3 h, when black formazan crystals appeared at the bottom of the wells, the medium was removed, and formazan was dissolved in dimethyl sulfoxide (DMSO). Absorbance was read on a plate reader (Spectra Max iD3, Molecular Devices, San Jose, CA, USA) at 570 nm. Cell viability was determined by dividing the absorbance of the experimental wells by the absorbance of the vehicle control wells × 100%. Three separate repeats of the experiment were performed. Data from cytotoxicity tests were subjected to one-way analysis of variance, followed by Dunnett’s test using GraphPad Prism 9.0 software (GraphPad Software Inc., San Diego, CA, USA). Values of *p* < 0.01 were considered statistically significant.

### 2.8. UPLC-MS/MS

Regardless of the above procedure, sample solutions of each of the tested coxibs, obtained after hydrolysis, were analyzed by UPLC-MS/MS to identify degradation products and determine possible drug degradation pathways. Solutions were analyzed using a Waters UPLC-MS/MS system, consisting of a Waters UPLC (Waters, Milford, MA, USA) coupled to a Waters mass spectrometer (electrospray ionization mode ESI-tandem quadrupole). Chromatographic separation was conducted on the UHPLC Cortecs T3 (2.1 × 100 mm, 1.7 μm) column, maintained at 40 °C. The following gradient of mobile phase was used: 0.1% ammonium formate in MilliQ water (A)/0.1% ammonium formate in acetonitrile (B): 0–10 min/5–50% B, 10–13 min/50–99% B, 13–15 min/99% B, 15–15.01 min/99–5% B, 15.01–20 min/5% B (+5 min 5% B post-time). Chromatograms were recorded in the 190–500 nm spectrum range, by a Waters DAD detector. The MS detection settings of the mass spectrometer were as follows: positive ion polarity, fragmentor voltage 120 V, nozzle voltage 1000 V, capillary voltage 3500 V, skimmer 65 V, octopole 750 V, gas flow 10 L/min, gas temperature 300 °C, nebulizer gas pressure 35 pisg, sheath gas flow 12 L/min, sheath gas temperature 350 °C, and collision energies 20 and 40 eV. The MS and MS/MS data were registered in a scan mode ranging from 70 to 1700 *m*/*z*, with a scan rate of 3 spectra/s.

## 3. Results and Discussion

The stability of the drug in various environmental conditions is of great importance for determining the effective dose of each preparation, which translates into a therapeutic effect. The obtained results of substance stability tests also affect the correct determination of storage conditions, and the use of appropriate excipients. Studies on the degradation products and hepatotoxicity tests of drug substance indicate the potential consequences of the use of drugs if they are not properly stored and/or used. The obtained results enables characterizing and comparing the behavior of drugs, on the example of selected coxibs, in various environmental conditions.

The decomposition of the tested coxibs, i.e., celecoxib, cimicoxib, firocoxib, etoricoxib and robenacoxib, in different environments (acidic and alkaline conditions: HCl and NaOH, water, buffer solutions with pH 2.0, 7.0 and 9.2) at different temperatures was analyzed chromatographically, and the obtained results are summarized in Tables S1–S3 (see Supplementary Material).

Next, we designated the basic kinetic parameters of the degradation reaction for five tested coxibs exposed to various environmental factors. The main kinetic parameters determining the course of the reaction are the reaction rate constant k and the half-life t_0.5_, the time after which the concentration of the substance will decrease by half [39]. In order to determine the order of reactions, based on the results of the percentage content of the substance at specific time points, dependence curves ln[%]_t_ = −kt + ln[%]_0_ were drawn. The obtained data enables concluding that the occurring reactions follow first-order kinetics. The calculated values of the reaction rate constants k and the times t_0.5_ and t_0.1_ for 23, 70 and 120 °C were shown in Table 1.

All tested drugs degraded faster in acidic than in alkaline or neutral environments, regardless of the tested temperature. The values of the reaction rate constant k increase with increasing temperature for all drugs, and t_0.5_ and t_0.1_ changed inversely. The tested coxibs were degraded to varying degrees under the same conditions, as evidenced by variable kinetics parameters (Table 1). Generally, the calculated values of k_alkaline_ < k_acidic_, and E_a(alkaline)_ > E_a(acidic)_. CIM is the most stable drug of all tested coxibs for any temperature and environment variant. In the acidic environment (1 M HCl) after 130 h at the temperature of 120 °C, 5.78% were left. This is also confirmed by the value of the reaction rate constant k, for CIM under these conditions it is 0.0219 h^−1^. In the case of 120 °C and buffer solution pH 7.0, only approximately 30% was degraded after 130 h of incubation (t_0.5_ = 256.8 h). In contrast, ROB is the drug that degrades the fastest in an acidic environment, regardless of the incubation temperature. At 120 °C, it was completely degraded after only 2 h. Summing up, it can be concluded that all substances are relatively stable and resistant to changing environmental conditions, as evidenced by the t_0.5_ and t_0.1_ values. At the same time, lower stability of drugs in acidic then in alkaline solutions was observed. Then, we compared the determined values of k, E_a_, ∆H*, ∆S* and logP (calculated using MLOGP software ver.1.1.2 [40]) for all analyzed substances (Table 2).

The calculated kinetic and thermodynamic parameters differ for respective drugs, and concern changes in the temperature during the hydrolysis processes, concentrations of environmental reagents and the nature of the tested compound. If the k values decrease and the times t_0.5_ and t_0.1_ increase, the stability of a given compound increases, i.e., the rate of its degradation in a given environment decreases. The values of entropy changes (∆H), determined at elevated temperature are positive, and at the same time the values of entropy changes (∆S) are negative, so the course of the reaction is more influenced by ∆S than ∆H. The Gibbs free energy ∆G takes more positive values when the reaction absorbs heat or reduces the entropy of the system. If ∆S < 0 and ∆H > 0, then ∆G > 0 and the reaction is non-spontaneous, i.e., it is necessary to provide additional energy (endothermic reaction). During them, the enthalpy of the system changes (products have more free energy than substrates). When dissolving a compound in water, the energy needed to dissolve it may come from this substance, which can absorb or give out heat after dissolution, or it may be taken from the reaction environment in the form of, e.g., thermal energy. The environment (acidic, neutral or alkaline) and heat acting on a drug result in the decomposition of the given compound. For such reactions, the enthalpy change Is positive (∆H*). When we compare the rates of two reactions with similar mechanisms, it can be assumed that the entropic effects will be comparable, i.e., the difference between the values of E_a_ and H will be small (negligible).

The lowest values of the designated parameters were found for CIM, and the highest for ROB. The values of E_a_, ∆H*, ∆S* for the tested drugs were observed to increase from 20.52 to 34.88 kJ/mol, from 17.25 to 31.61 kJ/mol, and from −80.43 to −43.89 J/mol∙K, respectively. At the same time, an increase in logP (lipophilicity parameter) from 2.18 to 4.47 was observed, from the most lipophobic CIM to the most lipophilic ROB. The results of the conducted experiments enables concluding that the stability of the tested coxibs decreases with an increase in their lipophilic character. For the most hydrophilic drug CIM, the calculated thermodynamic parameters are the lowest; and for the most hydrophobic ROB, they are the highest.

Chemometrics is a tool that uses mathematical, statistical and other formal logic methods to design or select optimal measurement procedures, and to extract the most relevant qualitative or quantitative information by analyzing chemical data [41].

**Figure 2:**
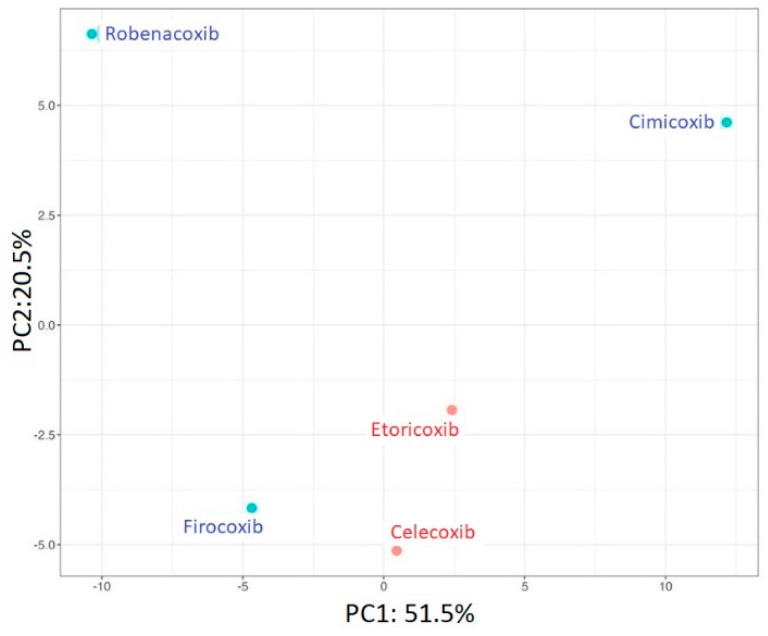
Results of PCA analysis; projection of individuals where human-used drugs are marked red, and veterinary-used drugs are colored blue.

In the next stage of our work, the results obtained after the degradation processes of the analyzed coxibs were subjected to chemometric analysis. PCA was performed to investigate and visualize the similarities and dissimilarities of investigated coxibs. Generally, PCA is an unsupervised multivariate technique that linearly transforms data into new orthogonal variables called principal components (PCs). Plotting data in 2D space created by PCs provided information about grouping tendency, including outlier detection. Here, presented in Figure 2, the two first PCs covered 72% of the information in the data matrix. Human-used ETO and CEL are the most similar among the studied molecules. On the other hand, veterinary-used drugs do not form a coherent group and differ significantly from each other.

**Figure 3:**
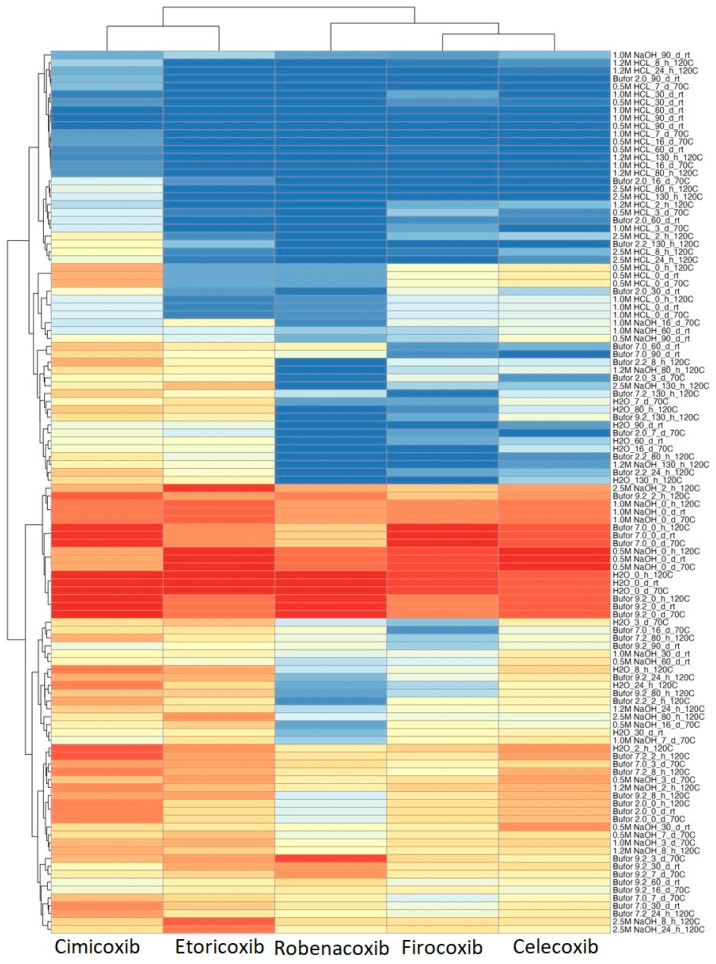
Results of HCA analysis presented as a heatmap. Labels of experimental conditions should be read, environment, then time in hours (h) or days (d), the temperature in Celsius degrees or room temperature (rt).

Tables S1–S3 refer to coxib degradation in several conditions that vary in temperature, pH, and time. A heatmap was performed to make the data coming from Tables S1–S3 more accessible to read and interpret (Figure 3). Conditions where only a small percentage of the substance remained, were marked blue, indicating significant degradation of the tested coxib. Opposite samples with a high percentage of coxib remained marked with red, indicating the high stability of investigated substances. Additionally, HCA was performed to investigate and visualize the similarities and dissimilarities of investigated coxibs in terms of their stability.

Generally, the structure of the HCA heatmap gives a quick overview and straightforward interpretation of which conditions differentiate the tested coxibs the most. In the case of HCA, two groups are formed, overlapping the use in humans or animals. All investigated coxibs degraded to a large extent at acidic pH regardless of temperature. Among tested substances in acidic conditioning, CIM showed the highest stability. A large percentage of the substance remained in the alkaline environment, as evidenced by the experiments with adding 0.5 and 1.0 M NaOH solutions. Regardless of the chemical structures investigated, coxibs remained largely undegraded. The main factors that primarily differentiated veterinary and human drugs were their lower stability in buffers at pH 7.2 and 9.0, respectively. On the other hand, ROB shows the lowest stability, especially in acidic and neutral, but also alkaline conditions.

The liver is the main organ responsible for the metabolism of xenobiotics, thanks to the multitude of enzyme systems, anatomical location and abundant vascularization. Chemical-induced hepatotoxicity is one of the most common causes of liver damage, and a huge challenge for both clinicians and pharmaceutical industry, especially due to the multitude of mechanisms by which it can occur [42,43]. Hepatic biotransformation of drugs leads to a change in a significant part of them from lipophilic to hydrophilic form, which results in the formation of water-soluble products, which are then excreted in the urine or bile. Basically, the treatment of the hepatotoxic effects of drugs comes down to discontinuation of therapy. Determination of the cytotoxicity of biologically active substances against cells in in vitro cultures is therefore a very important step in generally understood toxicological studies, necessary to determine the proper action and safety of the drug [40].

**Figure 4:**
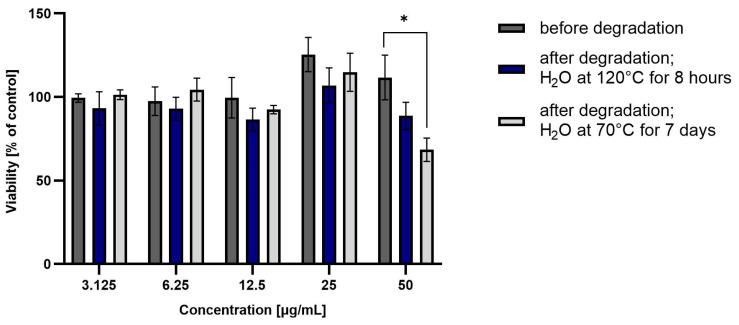
Viability of HepG2 cells incubated in the presence of ROB before and after degradation at different concentrations (3.125 to 50 µg/mL) for 48 h. Cell viability was determined using the MTT assay. The graphs represent the number of viable cells expressed as a percent of the control (cells not treated with compounds) ± SD (*n* = 3). * *p* < 0.01.

**Figure 5:**
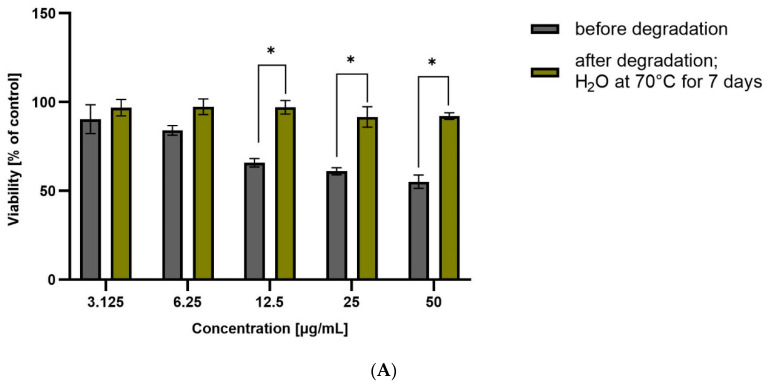

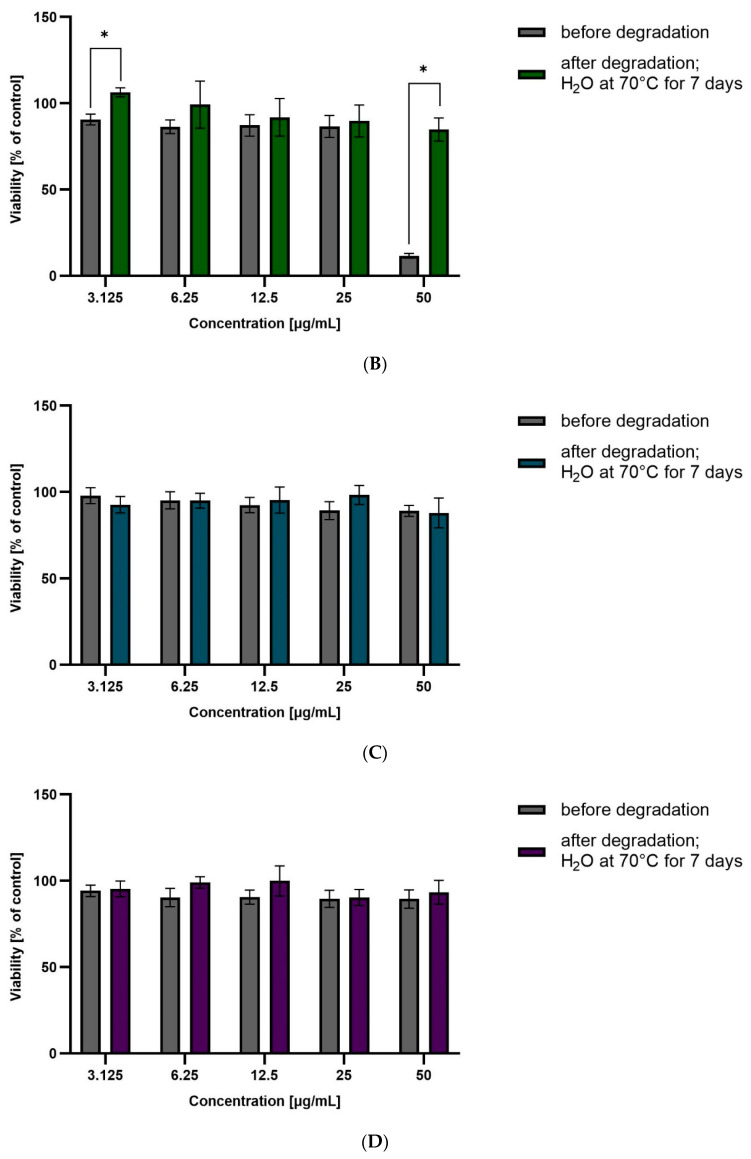
Viability of HepG2 cells incubated in the presence of CEL (**A**), CIM (**B**), ETO (**C**), FIR (**D**), before and after degradation at different concentrations (3.125 to 50 µg/mL) for 48 h. Cell viability was determined using the MTT assay. The graphs represent the number of viable cells expressed as a percent of the control (cells not treated with compounds) ± SD (*n* = 3). * *p* < 0.01.

Taking into account the obtained results of coxib degradation, it was decided to additionally perform a cell metabolic activity test (MTT). Each experiment was performed in triplicate. HepG2 cell viability was incubated in the presence of test drugs before and after degradation at various concentrations (from 3.125 to 50 µg/mL) for 48 h, and then the results are summarized in Figure 4 and Figure 5.

ROB after degradation in water at 70 °C for 7 days showed significant hepatotoxicity at the highest tested concentration (approximately 60% viability). This correlates with the high degradation of the drug, and the likely formation of a more hepatoxic product(s). For CIM and CEL, hepatotoxicity of the non-degraded drug can be observed at the highest concentrations (approximately 50% and 10% viability, respectively). Degraded samples show lower toxicity, suggesting that as the amount of active substance is reduced, the forming degradation products are less toxic than the parent compound.

As mentioned above, degradation of the analyzed substances in acidic environment revealed the presence of several new products. The mass fragmentation pathway of analyzed drugs was established using UPLC-MS/MS data acquired in the ESI positive ionization mode. The recorded chromatograms for the standard substance solutions and samples after degradation in 0.5 M HCl revealed several additional peaks (apart from the main peak from standard substance). The best possible molecular formulas of the fragments were determined using the elemental composition calculator. It has been observed that the degradation process mainly affects heteroatoms or carbon atoms in close proximity to heteroatoms, and involves bond cleavage, oxidation, hydration, hydrolysis and defluoration.

Based on previous results, we focused our attention on two of the tested compounds, namely CIM and ROB. These are substances characterized by the highest and the lowest stability in the analyzed conditions, respectively. The obtained mass spectra of analyzed compounds formed as a result of protonated drug hydrolysis, leading to few product ions. Their further fragmentation led to the formation of the main ion products. Under the analyzed conditions, no clear enough quantitative changes were registered during the degradation of cimicoxib, which would enable the correct analysis of the emerging products. Additionally, possible degradation products of firocoxib were included, as a third veterinary drug. The main structures with observed *m*/*z* values are shown in Table 3 and Table 4. In turn, Table 5 presents the proposed fragmentation pathways of robenacoxib degradation products.

As part of our study, all obtained results were analyzed and compared with similar research available in the literature. We noticed that Adhikari et al. conducted a degradation study in the environment of 0.3 M HCl at 105 °C for 4 h, 1 M formic acid at 105 °C for 7 days, 1 M perchloric acid at 105 °C for 0.5 h and 1 M phosphoric acid at 105 °C for 16 h. This manuscript also presents degradation process under the influence of an alkaline environment by incubation in 1 M NaOH solution at 50 °C for 7 days, 1 M ethylamine at 105 °C for 7 days and 1 M triethylamine at 105 °C for 7 days. After 4 h of incubation in 0.3 M HCl for FIR degradation, approximately 7.4% was observed. A similar level was obtained for 105 °C in 1 M phosphoric acid after only 30 min [37]. Comparatively, our data show approximately 50% degradation of FIR after 2 h of incubation in buffer pH 2.0 at 120 °C. Based on the results of forced degradation, FIR was found to be quite stable under alkaline, and less stable under acidic conditions. In addition, a total of six major FIR degradation products were observed, and two of them were isolated and purified to confirm their chemical structure. CEL is an anti-inflammatory drug whose fate in surface waters is unknown. Therefore, Jiménez et al. conducted an experiment under forced biological, photochemical and thermal conditions to determine its persistence and degradation products in river water. The results suggest that CEL dissolved in river water is not biodegradable, while it is minimally affected by sunlight or high temperature (70 °C). Only irradiation at 254 nm promotes its complete degradation. CEL was degraded approx. 3% over 36 weeks when water was kept at room temperature, and exposure to sunlight was partially limited. Eleven degradation products were detected, and the structures of nine of them were unambiguously proposed [36]. Vora et al. performed a degradation of etoricoxib in 1 M HCl, 1 M NaOH and hydrogen peroxide, in a water bath at 80 °C for 4 h. Photolytic and thermal degradation of the substance was performed by exposing to short wavelength light (254 nm) and high temperature (105 °C) for 4 days. The amount of etoricoxib gradually decreased with heating time at 80 °C in 1 M HCl; more than 20% had degraded after 3 h. Similar results were obtained during alkaline degradation; after 2 h of incubation at 80 °C in 1 M NaOH solution, 27% of etoricoxib was degraded [31]. Matthews et al. developed a method for the assay of etoricoxib in human plasma. During the experiments, etoricoxib was found to be sensitive to UV light and highly fluorescent products were formed. The major photolysis products were further isolated, and their structures were elucidated by ^1^H NMR and HPLC-NMR. The results indicate that etoricoxib undergoes a photocyclization leading to the formation of two main isomeric products. The proposed reaction scheme is analogous to the cyclization of rofecoxib, which is known to undergo stilbene-phenanthrene photocyclization upon UV irradiation [44,45]. In addition, cases of serious hepatic reactions, including fulminant hepatitis (sometimes fatal), hepatic necrosis, hepatic failure (sometimes fatal or requiring transplantation), have been reported in the registration studies of celecoxib. Where the time period prior to the event was known, most serious hepatic adverse events were found to develop within one month of starting celecoxib treatment [46].

Taking into account the results of other research teams presented above and the results of our analyses, we would like to draw attention to the diverse behavior of drugs from the coxib group. The available literature on coxibs often states that they are effective and safe, especially when compared to some other NSAIDs [47]. However, attention should be paid to the processes occurring tin solutions, and products formed as a result of their degradation of the active substance to note that such generalization are inadvisable. As we know, some of these substances have already been withdrawn from treatment due to their harmful effects on the body. In addition to the proven side effects of rofecoxib and valdecoxib (suspected of causing heart attacks or strokes, which has resulted in increased mortality in patients with heart disease), other drugs in this class may behave differently depending on the environment (e.g., pH at the site of drug release), including cytotoxicity. This problem affects every organism, human or animal, to which a specialist prescribes therapy with a selected drug containing coxib. Our attention was therefore focused on other substances about which there are few studies available, and which are commonly used in veterinary medicine, especially in the treatment of rheumatic ailments or joint dysplasia in dogs and horses. Our observations indicate that robenacoxib in particular was characterized by rapid degradation in the aqueous environment, resulting in the formation of a product or products showing toxicity to liver cells. Based on the proposed structures, it can be assumed that the hepatotoxicity of the mixture of robenacoxib degradation products may be caused by the products RP-3 and RP-4, having catechol groups. As is known, the presence of such formations may cause the formation of reactive oxygen species and reactive quinone groupings during metabolism, capable of reacting, for example, with cellular proteins. In the case of both of these products, an N-substituted 2-aminophenol moiety appears in the structure, which can be metabolized to the reactive 6-iminocyclohexa-2,4-dien-1-one. Therefore, further researches covering a broader spectrum of potentially toxic effects on the body are necessary. The results presented in this paper will constitute the basis for further analyzes in this direction.

## 4. Conclusions

Summarizing, the presented analytical procedure can be successfully used to study quantitative changes in the degradation process of the tested coxibs in solutions. The obtained results indicate that stress conditions affect the analyzed coxibs in different ways, despite the similar chemical structure of the molecules of these compounds. All the tested substances turned out to be relatively stable and resistant to changing environmental conditions, as evidenced by the calculated kinetic and thermodynamic parameters of the processes taking place. Some differences are noticeable in the rate of the drug substance degradation process, and in its hepatotoxicity. Cimicoxib was the most stable, while robenacoxib was the least stable. The high degree of robenacoxib degradation likely results in the formation of more hepatotoxic product(s), while cimicoxib samples show much lower toxicity, suggesting that the resulting degradation products are less toxic than the parent compound. The results of our research indicate a real threat that may result from pharmacotherapy if a given drug is used for too long or unnecessarily.

## Figures and Tables

**Table 1:** Kinetic parameters describing degradation process of five coxibs in various conditions.

Substance	Environment	k [h^−1^]			t_0.5_ [h]			t_0.1_ [h]			E_a_ [kJ/mol]
		23 °C	70 °C	120 °C	23 °C	70 °C	120 °C	23 °C	70 °C	120 °C	
ROB	1 M HCl	nd	nd	nd	nd	nd	nd	nd	nd	nd	nd
	0.5 M HCl	nd	nd	nd	nd	nd	nd	nd	nd	nd	nd
	H_2_O	2.51 × 10^−3^	13.50 × 10^−3^	85.10 × 10^−3^	276.1	51.3	8.1	42.0	7.8	1.2	34.88
	0.5 M NaOH	0.84 × 10^−3^	5.24 × 10^−3^	1.19 × 10^−2^	828.9	132.3	58.2	126.0	20.1	8.8	26.44
	1 M NaOH	0.95 × 10^−3^	6.75 × 10^−3^	13.60 × 10^−3^	731.0	102.7	51.0	111.1	15.6	7.7	26.22
	buffer pH 2.0	4.43 × 10^−3^	44.30 × 10^−3^	846.00 × 10^−3^	156.4	15.7	0.8	23.8	2.4	0.1	51.98
	buffer pH 7.0	0.65 × 10^−3^	5.67 × 10^−3^	19.50 × 10^−3^	1069.4	122.2	35.5	162.5	18.6	5.4	33.74
	buffer pH 9.2	0.24 × 10^−3^	1.30 × 10^−3^	27.60 × 10^−3^	2875.5	533.1	25.1	436.9	8.1	3.8	46.96
CIM	1 M HCl	3.11 × 10^−3^	3.78 × 10^−3^	13.70 × 10^−3^	222.8	183.3	50.6	33.9	27.9	7.7	14.82
	0.5 M HCl	1.68 × 10^−3^	4.97 × 10^−3^	6.72 × 10^−3^	412.5	139.4	103.1	62.7	21.2	15.7	13.68
	H_2_O	0.39 × 10^−3^	1.84 × 10^−3^	3.08 × 10^−3^	1776.9	376.6	225.0	270.0	57.2	34.2	20.52
	0.5 M NaOH	0.22 × 10^−3^	0.89 × 10^−3^	2.52 × 10^−3^	3135.7	777.8	275.0	476.5	118.2	41.8	24.16
	1 M NaOH	0.87 × 10^−3^	2.70 × 10^−3^	3.52 × 10^−3^	801.2	256.7	196.9	121.7	39.0	29.9	13.91
	buffer pH 2.0	0.77 × 10^−3^	2.29 × 10^−3^	3.73 × 10^−3^	904.7	302.6	185.8	137.5	48.0	28.2	15.73
	buffer pH 7.0	0.17 × 10^−3^	1.20 × 10^−3^	2.60 × 10^−3^	3982.8	577.5	266.5	605.2	87.8	40.5	26.67
	buffer pH 9.2	0.40 × 10^−3^	2.05 × 10^−3^	3.23 × 10^−3^	1745.6	338.0	214.6	265.2	51.4	32.6	43.77
FIR	1 M HCl	1.95 × 10^−3^	15.00 × 10^−3^	33.00 × 10^−3^	355.4	46.2	21.0	54.0	7.0	3.2	28.04
	0.5 M HCl	1.96 × 10^−3^	12.00 × 10^−3^	31.50 × 10^−3^	353.6	57.8	22.0	53.7	8.8	3.3	27.58
	H_2_O	1.42 × 10^−3^	1.2.30 × 10^−3^	33.40 × 10^−3^	488.0	56.3	20.7	74.2	8.6	3.2	31.23
	0.5 M NaOH	0.63 × 10^−3^	1.59 × 10^−3^	10.60 × 10^−3^	1108.8	435.8	65.4	168.5	6.6	9.9	28.04
	1 M NaOH	1.01 × 10^−3^	1.97 × 10^−3^	13.90 × 10^−3^	686.1	351.8	49.9	104.3	53.5	7.6	25.99
	buffer pH 2.0	1.60 × 10^−3^	10.80 × 10^−3^	73.50 × 10^−3^	433.1	64.2	9.4	65.8	9.8	1.4	38.07
	buffer pH 7.0	0.82 × 10^−3^	4.56 × 10^−3^	7.08 × 10^−3^	844.1	152.0	97.9	128.3	23.1	14.9	21.43
	buffer pH 9.2	0.62 × 10^−3^	1.07 × 10^−3^	14.40 × 10^−3^	1126.8	647.7	48.1	171.2	98.4	7.3	31.23
ETO	1 M HCl	nd	nd	nd	nd	nd	nd	nd	nd	nd	nd
	0.5 M HCl	nd	15.00 × 10^−3^	54.60 × 10^−3^	nd	46.2	12.7	nd	7.0	1.9	28.98
	H_2_O	0.42 × 10^−3^	2.05 × 10^−3^	4.54 × 10^−3^	1665.9	338.0	152.6	253.1	51.4	23.2	23.71
	0.5 M NaOH	0.46 × 10^−3^	0.95 × 10^−3^	5.68 × 10^−3^	1503.3	729.5	122.0	228.4	110.8	18.5	24.85
	1 M NaOH	0.63 × 10^−3^	1.42 × 10^−3^	4.89 × 10^−3^	1107.0	488.0	141.7	168.2	74.2	21.5	20.29
	buffer pH 2.0	2.74 × 10^−3^	5.26 × 10^−3^	9.70 × 10^−3^	252.9	131.7	71.4	38.4	20.0	10.9	12.54
	buffer pH 7.0	0.25 × 10^−3^	0.69 × 10^−3^	3.62 × 10^−3^	2783.1	1004.3	191.4	422.9	152.6	29.1	26.44
	buffer pH 9.2	0.27 × 10^−3^	1.70 × 10^−3^	3.44 × 10^−3^	2576.2	407.6	201.5	391.4	61.9	30.6	25.30
CEL	1 M HCl	nd	nd	106.00 × 10^−3^	nd	nd	6.5	nd	nd	1.0	nd
	0.5 M HCl	4.09 × 10^−3^	30.40 × 10^−3^	85.10 × 10^−3^	169.4	22.8	8.1	25.7	3.5	1.2	30.09
	H_2_O	1.03 × 10^−3^	2.90 × 10^−3^	10.90 × 10^−3^	672.8	239.0	63.6	102.2	36.3	9.7	23.48
	0.5 M NaOH	0.34 × 10^−3^	2.02 × 10^−3^	5.70 × 10^−3^	2056.4	343.1	121.6	312.5	52.1	18.5	28.04
	1 M NaOH	0.76 × 10^−3^	2.00 × 10^−3^	17.10 × 10^−3^	910.6	346.5	40.5	138.4	52.7	6.2	30.78
	buffer pH 2.0	1.83 × 10^−3^	30.30 × 10^−3^	28.70 × 10^−3^	378.7	22.9	24.1	57.5	3.5	3.7	27.36
	buffer pH 7.0	1.29 × 10^−3^	2.14 × 10^−3^	8.19 × 10^−3^	537.2	323.8	84.6	81.6	49.2	12.9	18.24
	buffer pH 9.2	0.35 × 10^−3^	1.92 × 10^−3^	5.48 × 10^−3^	1974.4	360.9	126.5	300.0	54.8	19.2	27.13

nd—no data.

**Table 2:** Calculated parameters k, E_a_, ∆H*, ∆S* describing degradation process of five coxibs under neutral conditions, and logP values.

Compound	k [h^−1^]	E_a_ [kJ/mol]	∆H* [kJ/mol]	∆S* [J/mol·K]	∆G [kJ/mol]	logP
ROB	85.10 × 10^−3^	34.88	31.61	−80.43	63.22	4.47
CIM	3.08 × 10^−3^	20.52	17.25	−43.89	34.50	2.18
FIR	33.40 × 10^−3^	31.23	21.96	−55.88	43.92	2.25
ETO	4.54 × 10^−3^	23.71	20.44	−52.01	40.88	2.93
CEL	10.90 × 10^−3^	23.48	20.21	−51.42	40.42	3.18

**Table 3:** Proposed structures of firocoxib degradation products.

Compound	R_t_ [min]	*m*/*z*	Proposed Structure
Firocoxib	15.17	337.11	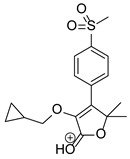
CP-1	7.17 7.33	243.07	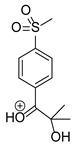
CP-2	9.65 9.98	355.12	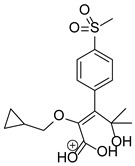
CP-3	9.71	283.06	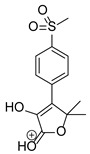
CP-4	15.85	387.11	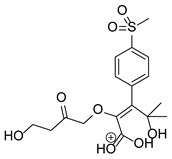

**Table 4:** Proposed structures of robenacoxib degradation products.

Compound	R_t_ [min]	*m*/*z*	Proposed Structure
Robenacoxib	19.59	328.09	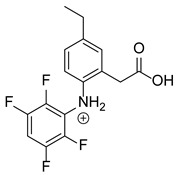
RP-1	1.79	166.09	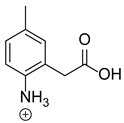
RP-2	5.07	180.10	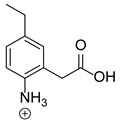
RP-3	19.84	282.09	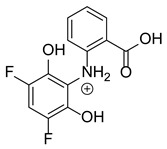
RP-4	19.96	310.08	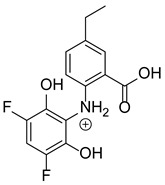
RP-5	20.23	314.08	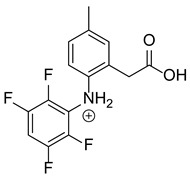

**Table 5:** Proposed fragmentation pathways of robenacoxib degradation products.

Product Name	Pathway
RP-1	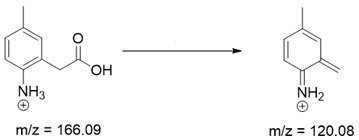
RP-2	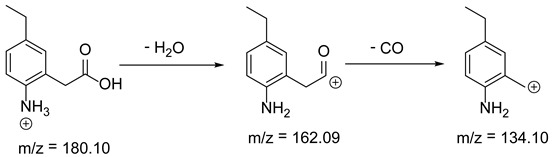
RP-3 and RP-4	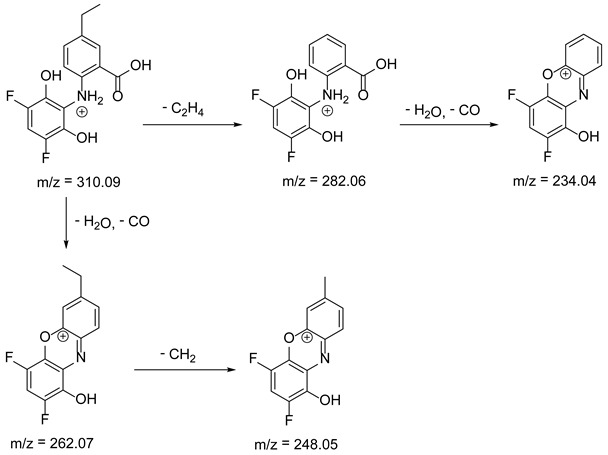
RP-5	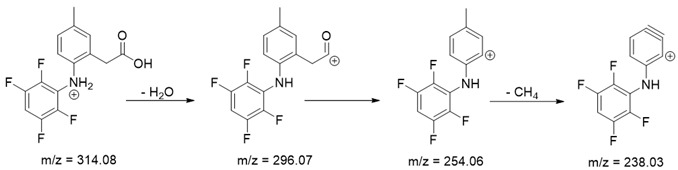
ROB	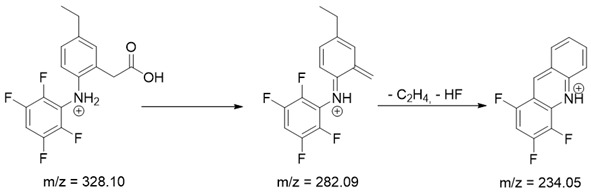

## Data Availability

Data are contained within the article.

## Supplementary Materials

The following supporting information can be downloaded at: https://www.mdpi.com/article/10.3390/pharmaceutics15112609/s1, Table S1: Results of coxibs degradation under different stress conditions at room temperature (23 °C); Table S2: Results of coxibs degradation under different stress conditions at 70 °C; Table S3: Results of coxibs degradation under different stress conditions at 120 °C.

## References

[1] Fu J.Y., Masferrer J.L., Seibert K., Raz A., Needleman P. (1990). The induction and suppression of prostaglandin Hz synthase. J. Biol. Chem..

[2] Paulus H.E., Whitehouse M.W. (1973). Nonsteroid anti-inflammatory agents. Annu. Rev. Pharmacol..

[3] Simmons D.L., Botting R.M., Hla T. (2004). Cyclooxygenase isozymes: The biology of prostaglandin synthesis and inhibition. Pharmacol. Rev..

[4] Regulski M., Regulska K., Prukała W., Piotrowska H., Stanisz B., Murias M. (2016). COX-2 inhibitors: A novel strategy in the management of breast cancer. Drug Discov. Today.

[5] Saxena P., Sharma P.K., Purohit P. (2020). Prostaglandins and other lipid mediators: A journey of celecoxib from pain to cancer. Prostaglandins Other Lipid Mediat..

[6] Park S.-I., Park J.-Y., Park M.-J., Yim S.-V., Kim B.-H. (2018). Effects of ojeok-san on the pharmacokinetics of celecoxib at steady-state in healthy volunteers. Basic Clin. Pharmacol. Toxicol..

[7] Walker C., Essex M.N., Li C., Park P.W. (2016). Celecoxib versus diclofenac for the treatment of ankylosing spondylitis: 12-week randomized study in Norwegian patients. J. Int. Med. Res..

[8] Zhang S., Zhang Y., Liu P., Zhang W., Ma J.-I., Wang J. (2016). Efficacy and safety of etoricoxib compared with NSAIDs in acute gout: A systematic review and a meta-analysis. Clin. Rheumatol..

[9] Kwiatkowska B., Majdan M., Mastalerz-Migas A., Niewada M., Skrzydło-Radomańska B., Mamcarz A. (2017). Status of etoricoxib in the treatment of rheumatic diseases. Expert panel opinion. Reumatologia.

[10] Bombardier C., Laine L., Reicin A., Shapiro D., Burgos-Vargas R., Davis B., Day R., Ferraz M.B., Hawkey C.J., Schnitzer T.J. (2000). Comparizon of upper gastrointestinal toxicity of rofecoxib and naproxen in patients with rheumatoid arthritis. N. Engl. J. Med..

[11] Arias L.H.M., González A.M., Fadrique R.S., Vázquez E.S. (2019). Gastrointestinal safety of coxibs: Systematic review and meta-analysis of observational studies on selective inhibitors of cyclooxygenase 2. Fundam. Clin. Pharmacol..

[12] Chandrasekharan N.V., Dai H., Roos K.L.T., Evanson N.K., Tomsik J., Elton T.S., Simmons D.L. (2002). COX-3, a cyclooxygenase-1 variant inhibited by acetaminophen and other analgesic/antipyretic drugs: Cloning, structure, and expression. Proc. Natl. Acad. Sci. USA.

[13] Pillans P.I., Ghiculescu R.A., Lampe G., Wilson R., Wong R., Macdonald G.A. (2012). Severe acute liver injury associated with lumiracoxib. J. Gastroenterol. Hepatol..

[14] Braun J., Baraliakos X., Westhoff T. (2019). Nonsteroidal anti-inflammatory drugs and cardiovascular risk–a matter of indication. Semin. Arthritis Rheum..

[15] Meek I.L., Van de Laar M.A.F.J., Vonkeman H.E. (2010). Non-steroidal anti-inflammatory drugs: An overview of cardiovascular risks. Pharmaceuticals.

[16] Sgambati S.A. (2005). Cardiovascular events associated with rofecoxib in a colorectal adenoma chemoprevention trial: Commentary. Dis. Colon Rectum..

[17] Cox S., Villarino N., Sommardahl C., Kvaternick V., Zarabadipour C., Siger L., Yarbrough J., Amicucci A., Reed K., Breeding D. (2013). Disposition of firocoxib in equine plasma after an oral loading dose and a multiple dose regimen. Vet. J..

[18] Bergh M.S., Budsberg S.C. (2005). The coxib NSAIDs: Potential clinical and pharmacologic importance in veterinary medicine. J. Vet. Intern. Med..

[19] Subhahar M.B., Singh J., Albert P.H., Kadry A.M. (2019). Pharmacokinetics. metabolism and excretion of celecoxib, a selective cyclooxygenase-2 inhibitor, in horses. J. Vet. Pharmacol. Ther..

[20] Kim T.W., Łebkowska-Wieruszewska B., Owen H., Yun H.I., Kowalski C.J., Giorgi M. (2014). Pharmacokinetic profiles of the novel COX-2 selective inhibitor cimicoxib in dogs. Vet. J..

[21] Kvaternick V., Malinski T., Wortmann J., Fischer J. (2007). Quantitative HPLC-UV method for the determination of firocoxib from horse and dog plasma. J. Chromatogr. B Anal. Technol. Biomed. Life Sci..

[22] Kongara K., Chambers J.P. (2018). Robenacoxib in the treatment of pain in cats and dogs: Safety, efficacy, and place in therapy. Vet. Med. Res. Reports..

[23] Giorgi M., Kim T.-W., Saba A., Rouini M.-R., Yun H., Ryschanova R., Owen H. (2013). Detection and quantification of cimicoxib. a novel COX-2 inhibitor. in canine plasma by HPLC with spectrofluorimetric detection: Development and validation of a new methodology. J. Pharm. Biomed. Anal..

[24] Jung M., Lees P., Seewald W., King J.N. (2009). Analytical determination and pharmacokinetics of robenacoxib in the dog. J. Vet. Pharmacol. Ther..

[25] Knych H.K., Stanley S.D., Arthur R.M., Mitchell M.M. (2014). Detection and pharmacokinetics of three formulations of firocoxib following multiple administrations to horses. Equine Vet. J..

[26] Donnell J.R., Frisbie D.D. (2014). Use of firocoxib for the treatment of equine osteoarthritis. Vet. Med..

[27] Jeunesse E.C., Schneider M., Woehrle F., Faucher M., Lefebvre H.P., Toutain P.-L. (2013). Pharmacokinetic/pharmacodynamic modeling for the determination of a cimicoxib dosing regimen in the dog. BMC Vet. Res..

[28] Morris T.H., Paine S.W., Zahra P.W., Li E.C., Colgan S.A., Karamatic S.L. (2020). Pharmacokinetics of carprofen and firocoxib for medication control in racing greyhounds. Aust. Vet. J..

[29] Tamizi E., Jouyban A. (2016). Forced degradation studies of biopharmaceuticals: Selection of stress conditions. Eur. J. Pharm. Biopharm..

[30] Baheti K.G., Shaikh S. (2011). Stability indicating RP-HPLC method for simultaneous estimation paractamol and etoricoxib in tablet formulation. Int. J. PharmTech Res..

[31] Vora D.N., Kadav A.A. (2009). Separation of etoricoxib and its degradation products in drug substance using UPLC TM. Eurasian J. Anal. Chem..

[32] Alzweiri M., Sallam M., Al-Zyoud W., Aiedeh K. (2019). Stability study of etoricoxib a selective cyclooxygenase-2 inhibitor by a new single and rapid reversed phase HPLC method. Symmetry.

[33] Venugopal S., Tripathi U.M., Devanna N. (2011). Validated reverse phase HPLC method for the determination of impurities in etoricoxib. J. Chem..

[34] Bapatu H.R., Maram R.K., Murthy R.S. (2015). Stability-indicating HPLC method for quantification of celecoxib and diacerein along with its impurities in capsule dosage form. J. Chromatogr. Sci..

[35] Chandana O.S.S., Ravichandrababu R. (2017). Stability indicating HPLC method for celecoxib related substances in solid dosage forms. Int. J. Res. Pharm. Sci..

[36] Jiménez J.J., Pardo R., Sánchez M.I., Muñoz B.E. (2018). Photochemical, thermal, biological and long-term degradation of celecoxib in river water. Degradation products and adsorption to sediment. J. Hazard. Mater..

[37] Adhikari S., Tian J., Rustum A.M. (2023). Comprehensive study on degradation profile of firocoxib and structural elucidation of its key degradation products. J. Pharm. Biomed. Anal..

[38] Gumułka P., Dąbrowska M., Starek M. (2020). TLC-densitometric determination of five coxibs in pharmaceutical preparations. Processe.

[39] Bełtowska-Brzezinska M. (2009). Podstawy Kinetyki Chemicznej (Fundamentals of Chemical Kinetics).

[40] Huveneers-Oorsprong M.B.M., Hoogenboom L.A.P., Kuiper H.A. (1997). The use of the MTT test for determining the cytotoxicity of veterinary drugs in pig hepatocytes. Toxicol. Vitr..

[41] Heberger K., Vekey K., Telekes A., Vertes A. (2008). Chemoinformatics—Multivariate Mathematical–Statistical Methods for Data Evaluation. Medical Applications of Mass Spectrometry.

[42] Björnsson E.S. (2016). Hepatotoxicity by drugs: The most common implicated agents. Int. J. Mol. Sci..

[43] Jaeschke H., Gores G.J., Cederbaum A.I., Hinson J.A., Pessayre D., Lemasters J.J. (2002). Mechanisms of hepatotoxicity. Toxicol. Sci..

[44] Matthews C.Z., Subramanian R., Woolf E.J., Foster N., Matuszewski B.K. (2004). Isolation and structural characterization of the photolysis products of etoricoxib. Int. J. Pharm. Sci..

[45] Woolf E., Fu I., Matuszewski B. (1999). Determination of rofecoxib, a cyclooxygenase-2 specific inhibitor, in human plasma using high-performance liquid chromatography with post-column photochemical derivatization and fluorescence detection. J. Chromatogr. B.

[46] Soni P., Shell B., Cawkwell G., Li C., Ma H. (2009). The hepatic safety and tolerability of the cyclooxygenase-2 selective NSAID celecoxib: Pooled analysis of 41 randomized controlled trials. Curr. Med. Res. Opin..

[47] Beales I.L.P. (2020). Selective COX-2 inhibitors are safe and effective. BMJ.

