# Supplementary material for: Comprehensive Assessment of the Stability of Selected Coxibs in Variable Environmental Conditions along with the Assessment of Their Potential Hepatotoxicity
Source: Pharmaceutics. 2023 Nov 9;15(11):2609. doi: 10.3390/pharmaceutics15112609 (PMC10674268; doi:10.3390/pharmaceutics15112609)
Supplement: Supplementary file 1 [file pharmaceutics-15-02609-s001.zip › pharmaceutics-2664800-supplementary.pdf]

pharmaceutics

Supplementary information

Comprehensive Assessment of the Stability of Selected Coxibs in Variable Environmental Conditions along with the Assessment of Their Potential Hepatotoxicity

Paweł Gumułka, Łukasz Pecio, Paweł Żmudzki, Krzesimir Ciura, Krystyna Skalicka-Woźniak, Monika Dąbrowska and Małgorzata Starek

Table S1. Results of coxibs degradation under different stress conditions at room temperature (23 °C).

Environment	Time [day]	Concentration [%]				
		ROB	CIM	FIR	ETO	CEL
1M HCl	0	7.63	34.45	32.67	4.88	36.04
	30	0.00	3.68	11.74	0.00	0.00
	60	0.00	0.00	1.98	0.00	0.00
	90	0.00	0.00	0.00	0.00	0.00
0.5M HCl	0	11.57	73.82	47.59	12.05	58.79
	30	0.00	8.22	7.72	0.00	3.09
	60	0.00	6.54	2.84	0.00	0.00
	90	0.00	0.00	0.00	0.00	0.00
H_2O	0	99.86	98.94	94.10	98.96	91.26
	30	16.36	51.81	51.26	56.65	56.67
	60	0.00	46.12	12.30	49.47	22.26
	90	0.00	42.57	0.00	40.32	9.87
0.5M NaOH	0	88.88	77.64	94.07	99.67	99.92
	30	50.72	66.29	58.86	62.54	82.35
	60	27.77	53.04	33.15	46.30	63.45
	90	14.60	48.19	24.38	36.82	48.28
1M NaOH	0	79.30	86.18	83.69	89.97	86.29
	30	38.93	51.04	41.28	55.46	59.26
	60	21.06	32.48	24.58	33.88	39.95
	90	10.23	13.31	9.55	23.28	16.67
buffer pH 2.0	0	36.30	84.29	65.26	66.57	73.75
	30	1.50	46.22	43.72	9.24	23.89
	60	0.00	32.78	6.49	0.00	5.28
	90	0.00	16.12	0.00	0.00	0.00
buffer pH 7.0	0	89.05	98.64	99.73	82.62	92.18
	30	59.96	83.58	43.39	70.92	59.01
	60	44.77	72.52	38.78	59.93	14.29
	90	21.99	67.79	16.94	48.25	0.00
buffer pH 9.2	0	98.57	99.89	84.89	87.72	91.03
	30	80.40	52.90	60.34	77.54	66.88
	60	65.98	44.73	41.99	55.67	53.22
	90	58.57	42.40	22.51	49.06	42.69

Table S2. Results of coxibs degradation under different stress conditions at 70 °C.

Environment	Time [day]	Concentration [%]				
		ROB	CIM	FIR	ETO	CEL
1M HCl	0	7.63	34.45	32.67	4.88	36.04
	3	0.00	32.69	11.11	0.00	0.00
	7	0.00	9.65	0.00	0.00	0.00
	16	0.00	8.06	0.00	0.00	0.00
0.5M HCl	0	11.57	73.82	47.59	12.05	58.79
	3	0.00	34.17	19.99	4.08	6.58
	7	0.00	17.76	0.00	0.00	0.00
	16	0.00	10.95	0.00	0.00	0.00
H_2O	0	99.86	98.94	94.10	98.96	91.26
	3	30.94	65.57	18.49	72.61	59.00
	7	10.37	51.55	11.89	64.78	43.29
	16	0.00	48.82	0.00	45.07	29.94
0.5M NaOH	0	88.88	77.64	94.07	99.67	99.92
	3	56.10	71.61	65.06	78.95	79.17
	7	42.48	65.10	59.42	72.75	64.79
	16	11.89	55.14	51.06	69.21	45.96
1M NaOH	0	79.30	86.18	83.69	89.97	86.29
	3	50.43	74.23	65.23	76.26	68.83
	7	22.52	44.45	52.79	63.27	61.26
	16	5.95	30.57	39.32	52.09	40.05
buffer pH 2.0	0	36.30	84.29	65.26	66.57	73.75
	3	1.50	51.65	40.87	49.58	8.32
	7	0.00	45.22	10.58	32.27	0.00
	16	0.00	34.93	0.00	8.85	0.00
buffer pH 7.0	0	89.05	98.64	99.73	82.62	92.18
	3	65.98	79.66	54.21	78.31	66.86
	7	36.08	73.48	34.79	67.89	52.69
	16	10.10	62.12	17.29	63.40	40.51
buffer pH 9.2	0	98.57	99.89	84.89	87.72	91.03
	3	95.05	74.71	67.69	79.89	56.39
	7	81.18	59.12	59.14	68.66	54.97
	16	59.92	45.42	56.22	58.19	43.50

Table S3. Results of coxibs degradation under different stress conditions at 120 °C.

Environment	Time [day]	Concentration [%]				
		ROB	CIM	FIR	ETO	CEL
1M HCl	0	7.63	34.45	32.67	4.88	36.04
	2	0.00	26.67	13.63	0.00	17.49
	8	0.00	22.63	2.33	0.00	7.66
	24	0.00	13.90	0.00	0.00	2.81
	80	0.00	8.35	0.00	0.00	0.00
	130	0.00	5.78	0.00	0.00	0.00
0.5M HCl	0	11.57	76.82	47.59	12.05	58.79
	2	0.00	55.95	16.68	6.74	22.53
	8	0.00	50.03	3.83	5.14	15.14
	24	0.00	43.52	0.00	3.25	7.63
	80	0.00	39.41	0.00	0.00	0.00
	130	0.00	32.09	0.00	0.00	0.00
H_2O	0	99.86	98.94	95.10	98.96	91.26
	2	58.93	92.10	69.02	81.86	81.53
	8	29.42	86.16	43.65	77.50	68.01
	24	12.96	85.46	24.77	66.88	52.87
	80	0.00	71.85	6.42	64.16	31.18
	130	0.00	66.25	0.00	54.86	22.14
0.5M NaOH	0	88.88	77.64	94.07	99.67	99.92
	2	77.40	75.05	70.55	73.76	79.58
	8	66.74	68.49	65.07	69.59	63.30
	24	54.62	64.09	62.62	62.87	54.30
	80	34.71	59.98	42.23	54.13	42.53
	130	0.00	55.99	23.72	47.65	20.28
1M NaOH	0	79.30	86.18	83.69	89.97	86.29
	2	56.19	83.53	69.66	73.76	74.95
	8	49.49	76.09	58.57	69.59	64.06
	24	26.71	69.29	47.65	62.87	51.98
	80	0.00	64.49	27.51	54.13	38.43
	130	0.00	55.94	0.00	47.65	9.35
buffer pH 2.0	0	36.30	84.29	65.26	66.57	73.75
	2	6.69	78.40	49.50	61.91	54.59
	8	0.00	76.17	35.84	54.64	31.44
	24	0.00	71.58	11.19	47.18	17.01
	80	0.00	61.92	0.00	41.43	7.42
	130	0.00	51.89	0.00	18.88	0.00
buffer pH 7.0	0	89.05	98.64	99.73	82.62	92.18
	2	63.79	92.57	87.96	78.69	79.64
	8	50.30	85.75	78.54	76.31	76.88
	24	42.84	79.36	63.52	61.71	58.79
	80	28.66	76.60	51.28	58.60	45.97
	130	7.06	70.42	39.71	51.63	31.80
buffer pH 9.2	0	98.57	99.89	84.89	87.72	91.03
	2	85.25	91.91	71.12	79.24	81.51
	8	32.91	78.91	60.95	78.86	74.79
	24	16.90	76.57	41.95	75.44	61.60
	80	10.87	71.72	26.19	70.11	56.04
	130	0.00	65.63	12.98	56.11	44.65
